# Optimal markers of treatment response to vasodilatory drugs in small vessel disease: An OxHARP trial analysis

**DOI:** 10.1177/17474930251360093

**Published:** 2025-07-10

**Authors:** Alastair J S Webb, Karolina Feakins, Amy Lawson, Catriona Stewart, James Thomas, Osian Llwyd

**Affiliations:** 1 Department of Brain Sciences, Imperial College London, London, UK; 2 Wolfson Centre for Prevention of Stroke and Dementia, University of Oxford, Oxford, UK; 3 Department of Basic and Clinical Neuroscience, King’s College London, London, UK; 4 School of Medicine, University of St. Andrews, St. Andrews, UK

**Keywords:** Small vessel disease, cerebrovascular reactivity, cerebral pulsatility

## Abstract

**Background and Aims:**

Vasodilating drugs targeting the endothelium could reduce long-term harms due to cerebral small vessel disease (cSVD) but there are no commonly accepted methods to measure short-term disease activity or drug response. In the OxHARP clinical trial, we determined the most sensitive physiological markers of treatment response to sildenafil versus placebo on either transcranial ultrasound (TCD) or magnetic resonance imaging (MRI), and their validity compared to disease severity and other measures of other physiological mechanisms.

**Methods:**

In the OxHARP double-blind, randomized, placebo-controlled crossover trial we measured aortic blood pressure, mean flow velocity (MFV), cerebral pulsatility, cerebrovascular conductance index (CVCi = MFV/aortic mean BP), cerebral perfusion (pcASL-MRI) and cerebrovascular reactivity to inhaled CO2 on TCD (CVR-TCD) and MRI in white (CVR-WM), gray (CVR-GM) and white matter hyperintensities (CVR-WMH). Effects of 3 weeks of sildenafil were compared to placebo. Validity of markers were determined by between-visit repeatability (intraclass correlation coefficient (ICC)); associations with CVR-TCD, CVR-WMH and CVR-GM; associations with other markers; the magnitude of response, and sensitivity, to sildenafil.

**Results:**

In 69 participants, repeatability was greatest for MFV, pulsatility, CVCi and CVR-WMH (ICC > 0.8), very good for CVR-TCD and GM-perfusion (ICC > 0.7), and good for CVR-GM (ICC > 0.6). CVR-TCD was associated with CVR on MRI (CVR-WMH: r^2^ = 0.12, p = 0.02; CVR-GM: r^2^ = 0.22, p = 0.001), while blood flow measures on TCD (MFV, CVCi) were associated with CVR-TCD and perfusion-MRI (all p < 0.05). All markers were associated with WMH volume and improved by sildenafil, but CVCi was most sensitive, requiring only 20 patients for a crossover trial at 80% power, compared to 26 for GM-perfusion or 84 for CVR-GM.

**Conclusions:**

Multiple markers were associated with cSVD, but no single marker reflected all physiological drug effects. CVCi and gray matter perfusion on MRI were the most sensitive markers of disease activity and drug response, although CVR indices may be more specific for endothelial dysfunction.

## Graphical abstract

**Graphical abstract:**
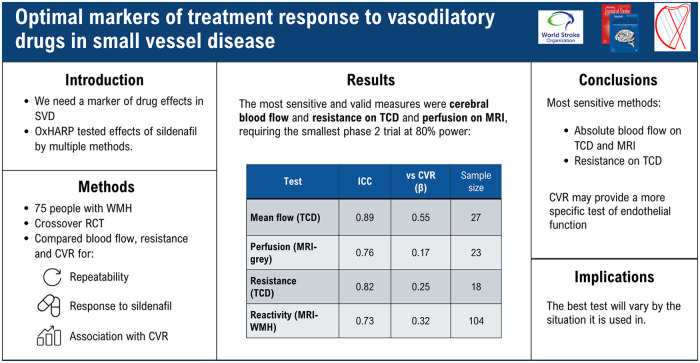

## Introduction

Cerebral small vessel disease (cSVD) causes 40% of all-cause dementia,^1^ 30% of ischemic stroke^2^ and 80% of intracerebral haemorrhage^3^ but has no specific intervention. The vasodilators^4^ cilostazol and isosorbide mononitrate (ISMN) increased cerebrovascular reactivity to inhaled carbon dioxide (CVR) in the LACI-1 trial^5^ and improved function (cilostazol) and cognition (ISMN or both) in LACI-2.^6^ PDE5 inhibitors improved cerebrovascular function in ETLAS-1^7^ and OxHARP,^8^ reversing the physiological dysfunction in cSVD, while PDE5 inhibition is a target in the POLARIS-AD phase 3 trial in Alzheimer’s disease.^9^

However, these trials mostly used fixed doses, have not included dose-finding studies and do not include markers of the physiological drug response. In contrast, established treatments for cardiovascular disease, including antihypertensives,^10^ anti-diabetic interventions^11^ or lipid-lowering therapies,^12^ use short-term rectification of physiological dysfunction to identify individuals who will benefit from treatment and to guide dose titration.^10^

cSVD evolves over many years^13^ and its management would benefit from short-term markers to guide treatment.^14^ An ideal marker of drug response should be easily measured, reproducible, associated with the disease, sensitive to treatment, and predict its long-term response, as for CVR.^15^ Although a blood test is ideal, limited cSVD-specific fluid biomarkers have been identified.^16^ On transcranial ultrasound (TCD) and magnetic resonance imaging (MRI), the OxHARP trial measured absolute blood flow, cerebrovascular resistance, and measures of cerebrovascular function (blood flow reactivity to inhaled CO_2_). We compared these hemodynamic measures to identify the most sensitive markers of response to sildenafil and their validity in comparison to disease severity and other mechanisms.

## Methods

### Study design

OxHARP was a double-blind, randomized, placebo-controlled, 3-way crossover phase 2 trial,^8^ that ran from 11 July 2019 to 6 December 2022 (Supplemental Figure S1). All procedures were performed at the Wolfson Center for Prevention of Stroke and Dementia. OxHARP was sponsored by the University of Oxford, approved by the UK Health Research Authority and South Central–Oxford C Research Ethics Committee (19/SC/0022), and registered with ClinicalTrials.org (NCT03855332). The study protocol,^17^ baseline features,^18^ and primary results^8^ have been published.

#### Participants

OxHARP recruited 75 participants with a previous cryptogenic or lacunar stroke or transient ischemic attack (TIA) requiring secondary preventive treatment, with mild to moderate white matter hyperintensities (WMH) evident on clinical brain imaging (Fazekas score on MRI or modified Blennow score on CT of 1–3 below 60 or 1–4 above 60). The full inclusion criteria have been reported previously.^17^ All participants without a contraindication were consecutively invited to join the MRI substudy. Participants who did not tolerate MRI were eligible to continue with TCD alone.

#### Procedures

Participants underwent assessments at baseline and at follow-up on each intervention (placebo, sildenafil, and cilostazol). At baseline and all follow-up visits, a standardized clinical and physiological assessment was performed, while MRI was performed at follow-up visits only. Following each visit, participants received treatment in randomized order with overencapsulated, double-blind medication. Each treatment lasted for 3 weeks, starting with thrice-daily placebo, thrice-daily sildenafil 25 mg, or twice daily cilostazol 50 mg, with the dose doubled after 1 week. Assessments were performed on the final day of treatment.

Physiological assessments were performed in a temperature-controlled laboratory (21–23°C) after 20 min supine rest, 30 min after observed administration of trial medication.

Middle cerebral artery flow velocity (MFV) was assessed by TCD via the transtemporal window during bilateral TCD monitoring with concurrent electrocardiogram (ECG), non-invasive blood pressure monitoring calibrated to an oscillometric brachial reading (FMS, Finometer Midi) and end-tidal carbon dioxide monitoring (etCO2, AD Instruments Gas Analyzer ML206). After 10 min rest, CVR was assessed during 2-min alternating periods of inhalation of medical air, 4% CO2 and 6% CO2, delivered via a respiratory circuit with a well-sealed, non-invasive ventilation mask. Aortic blood pressure was determined by radial artery applanation tonometry (SphygmoCor). After completion of physiological testing, CVR on MRI was assessed on multi-band blood oxygen level dependent (BOLD) MRI with whole brain acquisition every 800 ms and perfusion imaging with pseudo-continuous Arterial Spin Labeling (pcASL). Details of the imaging protocol have been reported previously.^17^ During CVR testing, participants breathed medical air or 6% CO2 (balance medical air) in 2 sets of 2-min periods, delivered by the same respiratory circuit used CVR-TCD.

#### Outcomes

Due to its strong association with SVD and high reproducibility,^19^ the primary outcome was Gosling’s middle cerebral artery pulsatility index (MCA-PI) on TCD, derived from the average of 3 manually measured peak systolic and 3 end-diastolic velocities, determined by 2 operators from the waveform envelope, blind to treatment allocation. The secondary outcome, as a widely used index of endothelial function,^5^ was CVR on TCD defined as change in mean flow velocity per mmHg change in etCO2 as the beta-coefficient from a linear model between etCO2 and mean MCA flow velocity during inhalation of medical air, 4% and 6% CO2, after correction for phase delay by cross-correlation with piecewise cubic Hermitte interpolation. Flow velocities were automatically measured from the exported waveform envelope with in house software, as described previously.^17^ For this analysis, additional outcomes included effects of each drug on aortic blood pressure, on absolute blood flow velocities in the middle cerebral artery (peak systolic, PSV; end-diastolic, EDV; mean flow, MFV) and on the relationship between them, estimated as the cerebrovascular conductance index (CVCi = MFV / aortic MBP).

BOLD-CVR images were pre-processed (MCFLIRT, B0-unwarping (BBR), high-pass temporally filtered at 300s and brain extracted). Regions of interest were defined by segmentation of T1 and FLAIR images (fMRIB Software Library, FSL: FAST/FIRST) into gray matter, WMH and normal appearing white matter (NAWM, FSL:BIANCA). CVR analysis was performed as described previously, expressing CVR as the average percentage change in BOLD response per mmHg change in etCO2 across all voxels in the regions of interest (NAWM and WMH). Calculations are reported in Supplemental Table S1.

#### Statistical analysis

Cerebrovascular reactivity was the primary marker of endothelial function targeted by sildenafil and cilostazol in OxHARP. We therefore compared alternative markers of treatment response that could provide surrogate measures of CVR, including blood pressure (systemic and aortic); mean cerebral blood flow velocity; cerebrovascular resistance, CVCi; resistance area product (RAP, inverse of the beta-coefficient between cerebral blood flow and blood pressure during diastole); cerebral perfusion on MRI (pcASL), for the mean, peak and trough values on TCD and each region-of-interest on MRI. To assess the validity of each index, we determined the consistency of associations with demographic risk factors (GLM, general linear models); repeatability between baseline and placebo for physiological measures, and consistency of treatment effects between placebo and sildenafil for MRI measures (intraclass correlation coefficient); associations between indices, particularly with measures of CVR; associations between change in each index on sildenafil versus placebo; the magnitude of change in each index as absolute mean and standardized by within-individual standard deviation. Bivariate correlations between key outcomes were calculated as Pearson’s correlation coefficients. General linear models were reported unadjusted or adjusted for age, sex and cardiovascular risk factors, including history of diabetes, smoking, hypertension, and dyslipidemia, and beta coefficients were determined both as unstandardized and standardized coefficients. Finally, we determined the predicted size of a phase 2 study to identify the equivalent effect seen in OxHARP with sildenafil, for either a parallel group or crossover designed trial. All analyses were performed in RStudio 2024.12.1 or Matlab 2018b.

The funder of the study had no role in study design or performance of the study.

## Results

Of 75 patients having physiological testing at baseline, 69 participants underwent physiological testing on placebo while 47 had an MRI scan (Table 1), with no differences between groups. The population was similar to observational TIA or minor stroke populations,^20^ with a median age of 70 and a majority having a history of hypertension, but with more men than women. Half the population had mild WMH on brain imaging and half had moderate-to-severe WMH.

**Table 1.** Demographic characteristics of patients included in the study.

Variables	All participants	MRI on placebo	TCD on placebo
N	75	47	69
Age	70 (7.7)	69 (7.5)	70 (7.8)
Male	59 (78)	37 (78)	55 (79)
Diabetes	5 (6)	3 (6)	4 (5)
Hypertension	57 (76)	34 (72)	52 (75)
Smoker	8 (10)	7 (15)	8 (11)
White	74 (98)	47 (100)	68 (98)
Alcohol (units)	9.5 (1 - 20)	9 (0.75 - 21)	9 (1 - 20)
WMH severity			
Mild	40 (53)	25 (53)	38 (55)
Moderate	20 (26)	11 (23)	17 (24)
Moderate-Severe	15 (20)	11 (23)	14 (20)
Systolic BP (mmHg)	127 (18)	124 (17)	125 (16)
Diastolic BP (mmHg)	70 (10)	69 (8.6)	70 (8.3)
Heart Rate (bpm)	62 (9)	61 (8.4)	61 (7.9)
Antihypertensives (median)	54 (72)	36 (76)	49 (71)
MoCA	27 (25 - 28)	27 (25.5 - 28.5)	27 (25 - 28)

Values are given as mean (SD) for normal variables, median (IQR) for skewed variables or number (%) for frequencies.

Repeatability between visits was excellent for physiological indices, including MFV, pulsatility and CVCi (intraclass correlation coefficient (ICC) > 0.8) and very good for CVR-TCD (ICC > 0.7). For consistency between placebo and treatment on imaging, this was excellent for CVR-WMH (ICC > 0.8), very good for GM-perfusion (ICC > 0.7) and good for CVR-GM (ICC > 0.6), but lower for CVR within normal appearing white matter. Overall, repeatability was greater when comparing baseline and placebo on TCD than comparing placebo and drug on MRI, and for absolute blood flow indices than for reactivity to carbon dioxide (Table 2). On MRI, there was greater repeatability for indices in the gray matter than either WMH or NAWM.

**Table 2.** Repeatability of indices of cerebrovascular dysfunction.

Variables	n	ICC	ICC CI	p-val	r^2^	p-val
PSV	69	0.864	(0.81 - 0.9)	< 0.001	0.45	<0.001
MFV	69	0.894	(0.85 - 0.92)	<0.001	0.41	<0.001
EDV	69	0.914	(0.88 - 0.94)	<0.001	0.38	<0.001
PI	69	0.89	(0.85 - 0.92)	<0.001	0.25	<0.001
CVCi mean	69	0.823	(0.74 - 0.88)	<0.001	0.31	<0.001
RAP	69	0.759	(0.65 - 0.84)	<0.001	0.078	<0.001
CVR-TCD MFV	69	0.776	(0.69 - 0.84)	<0.001	0.096	<0.001
CVR-TCD PSV	69	0.774	(0.69 - 0.84)	<0.001	0.12	<0.001
CVR-TCD EDV	69	0.724	(0.59 - 0.82)	<0.001	0.18	<0.001
CVR-WMH	47	0.847	(0.76 - 0.90)	<0.001	0.29	<0.001
CVR-NAWM	47	0.413	(0.077 - 0.63)	0.027	0.0064	0.067
CVR-GM	47	0.606	(0.38 - 0.75)	<0.001	0.048	0.0018
WMH perfusion	47	0.734	(0.58 - 0.83)	<0.001	0.2	<0.001
NAWM perfusion	47	0.745	(0.6 - 0.84)	<0.001	0.21	<0.001
GM perfusion	47	0.761	(0.62 - 0.85)	<0.001	0.24	<0.001

SBP = systolic blood pressure; DBP = diastolic blood pressure; PSV = peak systolic velocity; EDV = end-diastolic velocity; CVR = cerebrovascular reactivity; CVCi = cerebrovascular conductance index; ICC = intraclass correlation coefficient; CI = confidence interval. r^2^ and p values are given for the linear model between the two visits.

Age was associated with reduced EDV, increased pulsatility (Supplemental Tables S2 to S4), CVR in WMH and CVCi, after adjustment for other measures (Supplemental Table S5), while cognitive impairment was associated with a reduced MFV and increased CVCi. WMH volume was associated with reduced absolute blood flow on TCD (MFV, PSV and EDV), increased resistance (CVCi) and pulsatility and reduced CVR on TCD and MRI (Table 3, Supplemental Tables S2 to S5), with a trend to a reduction in gray matter perfusion (Supplemental Table S6). CVR-WMH was only associated with WMH volume after adjustment for age and cardiovascular risk factors.

**Table 3.** Association of indices of cerebrovascular dysfunction responsive to sildenafil, as reported in the primary results.

Variables	MFV r2	MFV p	CVCi r2	CVCi p	CVR-TCD r2	CVR-TCD p	CVR-WMH r2	CVR- WMH p	CVR- NAWM r2	CVR- NAWM p	GM perfusion r2	GM perfusion p-val
Age	0.082	0.014	0.038	0.095	0.016	0.292	0.18	0.003	0.035	0.205	0.002	0.758
Male	0.046	0.069	0.119	0.002	0.128	0.002	0.018	0.371	0.010	0.494	0.11	0.03
Diabetes	0.031	0.139	0.033	0.120	0.015	0.312	0.002	0.78	0.016	0.404	0.06	0.112
Hypertension	0.001	0.764	0.000	0.958	0.013	0.341	0.001	0.812	0.008	0.559	0.052	0.142
Smoker	0.474	0.183	0.452	0.309	0.613	0.057	0.445	0.872	0.582	0.834	0.526	0.523
Alcohol	0.313	0.683	0.306	0.221	0.273	0.984	0.528	0.377	0.358	0.115	0.566	0.603
Systolic BP	0.002	0.717	0.145	0.001	0.006	0.538	0.036	0.200	0.003	0.696	0.000	0.996
Diastolic BP	0.000	0.912	0.117	0.003	0.000	0.955	0.009	0.518	0.037	0.196	0.008	0.557
Stroke	0.030	0.145	0.017	0.259	0.081	0.017	0.071	0.071	0.009	0.532	0.006	0.635
MoCA	0.066	0.029	0.052	0.049	0.039	0.100	0.011	0.479	0.033	0.224	0.073	0.080
WMH volume	0.155	0.003	0.106	0.013	0.093	0.028	0.146	0.009	0.000	0.885	0.082	0.065

**Figure 1.**
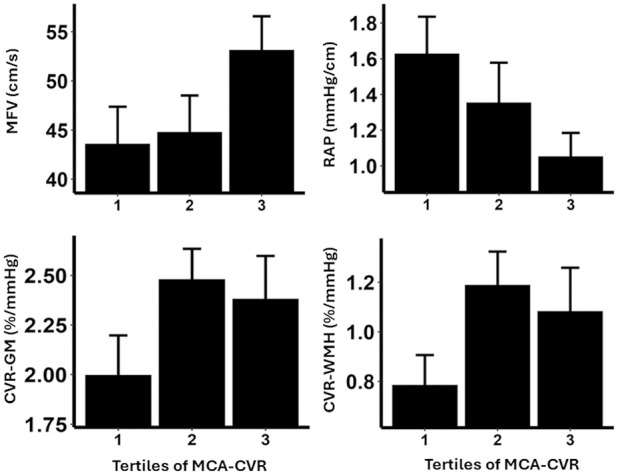
Relationships between middle cerebral artery cerebrovascular reactivity on transcranial ultrasound with mean flow velocity (MFV), cerebrovascular resistance (resistance area product, RAP), and cerebrovascular reactivity on MRI in gray matter (CVR-GM) and white matter hyperintensities (CVR-WMH).

CVR-TCD was associated with CVR on MRI in all tissues, with a stronger relationship with CVR in normal appearing white matter than WMH or gray matter (Figure 1, Table 4). CVR-TCD was also strongly associated with absolute cerebral blood flow velocities on TCD, and with cerebrovascular resistance (CVCi or RAP, Figure 1), but not with systemic blood pressure or cerebral pulsatility. In contrast, CVR-WMH and CVR-NAWM were not associated with absolute cerebral blood flow on either MRI or TCD (Table 4), although CVR in the gray matter was associated with cerebral perfusion (Supplemental Table S5). MCA velocities on TCD were associated with absolute cerebral perfusion on MRI. Bivariate associations between indices are shown in Supplemental Figure S2.

**Table 4.** Association of principal outcomes in OxHARP with markers of cerebrovascular endothelial dysfunction on placebo.

Variables	MFV CVR beta	MFV CVR r2	MFV CVR p	WMH CVR beta	WMH CVR r2	WMH CVR p	NAWM CVR beta	NAWM CVR r2	NAWM CVR p
Aortic Systolic BP	0.077	0.006	0.538	-0.192	0.036	0.200	-0.059	0.003	0.696
Aortic Diastolic BP	0.007	0.000	0.955	0.105	0.009	0.518	0.209	0.037	0.196
PSV	0.564	0.324	<0.001	0.059	0.003	0.696	-0.047	0.002	0.755
EDV	0.496	0.250	<0.001	0.158	0.025	0.289	0.068	0.005	0.652
MFV	0.553	0.312	<0.001	0.115	0.013	0.444	0.012	0.000	0.937
PI	-0.101	0.010	0.414	-0.270	0.063	0.088	-0.216	0.040	0.175
CVR-TCD PSV	0.899	0.807	<0.001	0.321	0.110	0.024	0.466	0.233	0.001
CVR-TCD EDV	0.939	0.881	<0.001	0.344	0.143	0.010	0.382	0.178	0.003
CVR-TCD MFV	-	-	-	0.319	0.122	0.018	0.429	0.222	0.001
CVCi MFV	0.492	0.249	<0.001	0.135	0.015	0.419	-0.034	0.001	0.840
RAP DBP	-0.376	0.142	0.003	0.083	0.007	0.599	0.210	0.042	0.19
CVR WMH	0.395	0.129	0.014	-	-	-	0.614	0.377	<0.001
CVR NAWM	0.496	0.202	0.002	0.614	0.377	< 0.001	-	-	-
CVR GM	0.395	0.125	0.016	0.623	0.388	< 0.001	0.742	0.550	<0.001
MRI perf WMH	0.316	0.075	0.080	0.105	0.011	0.510	0.063	0.004	0.697
MRI perf NAWM	0.227	0.038	0.216	-0.201	0.039	0.205	-0.049	0.002	0.763
MRI perf GM	0.172	0.022	0.353	-0.058	0.003	0.716	-0.049	0.002	0.761

Beta-coefficients are standardized. PSV = peak systolic velocity; EDV = end-diastolic velocity; CVR = cerebrovascular reactivity; CVCi = cerebrovascular conductance index.

Despite associations between CVR on MRI and TCD, there was no association between the sildenafil-placebo difference in CVR-TCD and the difference in CVR on MRI (Supplemental Table S7). There were only relatively weak associations between drug effects on CVR-TCD and drug effects on blood flow velocities, with a stronger association with effects on RAP. There were similarly no associations between the magnitude of effect of sildenafil on absolute markers of cerebral blood flow velocity on TCD with effects on cerebral perfusion on MRI (Supplemental Table S8). There was a lack of associations between indices for the effects of cilostazol (Supplemental Tables S9 to S10).

Sildenafil had a significant effect versus placebo on all markers except for PI, but the effect size differed (Table 5). When standardized by the within-individual standard deviation (SD), the strongest effect size on TCD was for MFV, RAP, and CVCi, with 57%, 49% and 71% of SD change respectively. On markers of perfusion on MRI, the strongest effect was in gray matter (61% of SD). Effect sizes for CVR were smaller, although still significant. This resulted in a smaller predicted sample size for trials using markers of resistance and absolute blood flow than CVR. However, markers of all physiological mechanisms resulted in an achievable sample size at 80% power, with the most sensitive markers on TCD being MFV, CVCi and RAP and the most sensitive marker on MRI being perfusion in the gray matter. CVR was less sensitive and required a larger trial. For a crossover designed trial, there is adequate power at 80% for trials smaller than 50 participants whereas parallel design trials required larger sample sizes due to greater between-individual variance. Overall, TCD was more sensitive than MRI. This pattern was consistent with different effect sizes, or methods of analysis (Supplemental Table S11).

**Table 5.** Differences in sensitivity to the effect of sildenafil versus placebo for each marker.

				Parallel			Crossover	
Variables	Mean on placebo	Mean change	SD of change	Standardized change	Sample size 80% power	Sample size 90% power	Sample size 80% power	Sample size 90% power
Aortic Systolic BP	120	-7.7	13	-0.58	61	81	25	32
Aortic Diastolic BP	74	-4.5	7.7	-0.58	59	78	25	33
PSV	78	7.5	11	0.69	64	86	19	25
EDV	32	2.3	5.8	0.40	173	231	52	69
Mean velocity	47	4.1	7.2	0.57	86	114	27	35
Pulsatility index	1.00	0.025	0.12	0.20	644	862	183	245
CVR-TCD PSV	8.50	0.82	3.7	0.22	198	264	162	216
CVR-TCD MFV	5.10	0.58	2.0	0.30	121	161	96	127
CVR-TCD EDV	6.60	0.77	2.5	0.31	118	158	85	113
CVCi (MFV/MBP)	0.53	0.078	0.11	0.71	45	60	18	23
RAP	1.40	0.53	0.47	-0.49	85	113	35	46
CVR WMH	1.00	0.078	0.28	0.27	374	500	104	138
CVR NAWM	0.76	0.053	0.20	0.26	163	218	114	152
CVR GM	2.20	0.17	0.52	0.33	148	198	76	101
MRI perf WMH	9.90	1.8	3.8	0.48	79	105	37	49
MRI perf NAWM	20	2.5	4.6	0.54	86	115	29	38
MRI perf GM	29	3.7	6.0	0.61	68	90	23	30

SD = standard deviation; PSV = peak systolic velocity; EDV = end-diastolic velocity; CVR = cerebrovascular reactivity; CVCi = cerebrovascular conductance index.

## Discussion

In this post hoc analysis of the OxHARP trial, sildenafil increased cerebral blood flow, reduced cerebrovascular resistance and increased cerebrovascular reactivity on both TCD and MRI. All measures were associated with severity of cSVD. However, while measures of blood flow on TCD were associated with blood flow on MRI, and CVR on TCD was associated with CVR on MRI, measures of blood flow on one modality were not associated with measures of CVR on the other modality. There were also no associations between the size of the response to sildenafil between different indices, except that the CVCi or RAP response to drug reflected the CVR response to drug. Finally, the magnitude of the treatment effect differed, with the most sensitive markers being cerebrovascular resistance on TCD (CVCi or RAP) and cerebral perfusion in the gray matter on MRI, thus requiring a smaller sample size for future trials.

Cerebral small vessel disease currently has no specific treatment.^21^ LACI-2^6^ suggested that isosorbide mononitrate, targeting endothelium-dependent vasodilatation, reduces cognitive decline at 1 year while cilostazol 100 mg twice daily reduces functional decline. However, these trials used specific drug doses, did not follow any dose-finding studies, and did not incorporate short-term physiological measures of the treatment effect. LACI-1^5^ measured CVR to carbon dioxide on MRI but did not compare the sensitivity or validity of the CVR response with other measures. Furthermore, CVR on MRI was insensitive in OxHARP and it is impractical for clinical dose titration. The TREAT-SVDs^22^ trial found no benefit of vasodilating antihypertensives on CVR on MRI, but included no measure of absolute cerebral perfusion. Similarly, PASTIS^23^ and ETLAS-1^7^ tested the effects of single doses of tadalafil on MRI-NAWM and TCD-MFV respectively, but neither demonstrated a significant effect.

An ideal short-term marker of physiological dysfunction in cSVD would be associated with: disease severity, other mechanisms of physiological dysfunction, other responses to treatment, and be highly sensitive. However, no single index in OxHARP fulfilled these criteria. Measures of blood flow on TCD were only associated with blood flow on MRI but not CVR, while CVR on TCD was only significantly correlated with CVR on MRI. However, CVR measures on TCD explained 10–25% of the variance on MRI, suggesting that TCD may provide a surrogate for cross-sectional MRI measures where MRI is not practical. Furthermore, CVCi and RAP could be used as surrogate markers of both absolute cerebral blood flow on TCD and CVR on TCD. Highly sensitive tests such as CVCi or RAP on TCD may be preferable in phase 2 trials screening multiple drugs or testing dose-response. Where TCD is not available, perfusion in the gray matter on MRI would be the most sensitive test. However, CVR may provide a more specific assessment of vasodilatory capacity and function.

The effect of sildenafil on multiple mechanisms demonstrates its effect at multiple sites in the cardiovascular system. It reduced central aortic pressure, absolute blood flow velocity in large cerebral vessels on TCD, perfusion in parenchymal small vessels on MRI, and responsivity of both large vessel flow and parenchymal perfusion to CO2. The greater sensitivity of CVCi and RAP may therefore reflect the effects of sildenafil on both blood pressure and cerebral blood flow. However, blood flow at each of these sites is not independent due to large vessel-small vessel cross-talk, with a reduction in small vessel resistance due to arteriolar vasodilatation resulting in increased proximal flow in cerebral blood vessels and the aorta.^24^

There are limitations to this study. First, this was a post hoc analysis. However, OxHARP was specifically designed to include multiple physiological methods to determine drug effects at multiple sites. No previous studies in cSVD have included such a comprehensive physiological approach.^5,7,22,23^ Second, the OxHARP trial had excess male participants, and results may differ in populations with a higher proportion of women. Third, MRI was only performed on drug and placebo and not at baseline and within-individual estimates of between-visit variance in MRI measures may be increased by the effect of sildenafil. Therefore, these estimates of repeatability have reduced validity. Fourth, the CVR paradigm on MRI used a “boxcar” design. This has the advantage of simplicity and easy translation but more advanced methods for control of CO2 (ramp designs, end-tidal forcing with a respiract) may be more precise, provide more detail regarding the dose-response, not be subject to “ceiling” effects of dosing at 6% and be more sensitive. Fifth, given the number of statistical comparisons, confidence in the statistical validity of any individual analysis would require an adjusted significance threshold of <0.001. Finally, the effect size for powering future trials is based upon the effect size of sildenafil in this trial, which may not be applicable to other drugs.

OxHARP was not designed to demonstrate a disease modifying effect on cSVD, but these results strongly suggest the need for further testing to determine effects on progression of cSVD and on clinical outcomes.^25^ In addition, studies are needed to identify the optimal dose, marker of treatment effect and population to treat prior to phase 3 clinical trials, as well as to test other vasodilatory targets (ISMN, PDE3, endothelin-1 antagonists) and other mechanisms (endothelial cell activation, neuroinflammation, extracellular matrix dysfunction).

## Conclusions

PDE5 inhibition improved cerebral blood flow and cerebrovascular reactivity, with comparable effects on TCD and MRI, but no single physiological marker was representative of all mechanistic targets. A comprehensive physiological assessment is therefore necessary for mechanistic studies, but highly sensitive measures (CVCI or RAP on TCD and GM-perfusion on MRI) can support optimization of drug doses for clinical trials and future clinical practice. These should be included in future phase 2 and phase 3 trials to support translation to clinical practice.

## Supplemental Material

sj-docx-1-wso-10.1177_17474930251360093 – Supplemental material for Optimal markers of treatment response to vasodilatory drugs in small vessel disease: An OxHARP trial analysis Supplemental material, sj-docx-1-wso-10.1177_17474930251360093 for Optimal markers of treatment response to vasodilatory drugs in small vessel disease: An OxHARP trial analysis by Alastair J S Webb, Karolina Feakins, Amy Lawson, Catriona Stewart, James Thomas and Osian Llwyd in International Journal of Stroke
