# Supplementary material for: Optimal markers of treatment response to vasodilatory drugs in small vessel disease: An OxHARP trial analysis
Source: Int J Stroke. 2025 Jul 10;21(1):100–9. doi: 10.1177/17474930251360093 (PMC12743133; doi:10.1177/17474930251360093)
Supplement: sj-docx-1-wso-10.1177_17474930251360093 – Supplemental material for Optimal markers of treatment response to vasodilatory drugs in small vessel disease: An OxHARP trial analysis [file sj-docx-1-wso-10.1177_17474930251360093.docx]

**SUPPLEMENTARY DATA**

**Optimal markers of treatment response to vasodilatory drugs in small vessel disease:**

**An OxHARP Trial analysis**

**Webb AJS, Lawson A, Feakins K, Stewart C, Thomas J, Llwyd O**

# Figure S1.A schematic of the trial design.


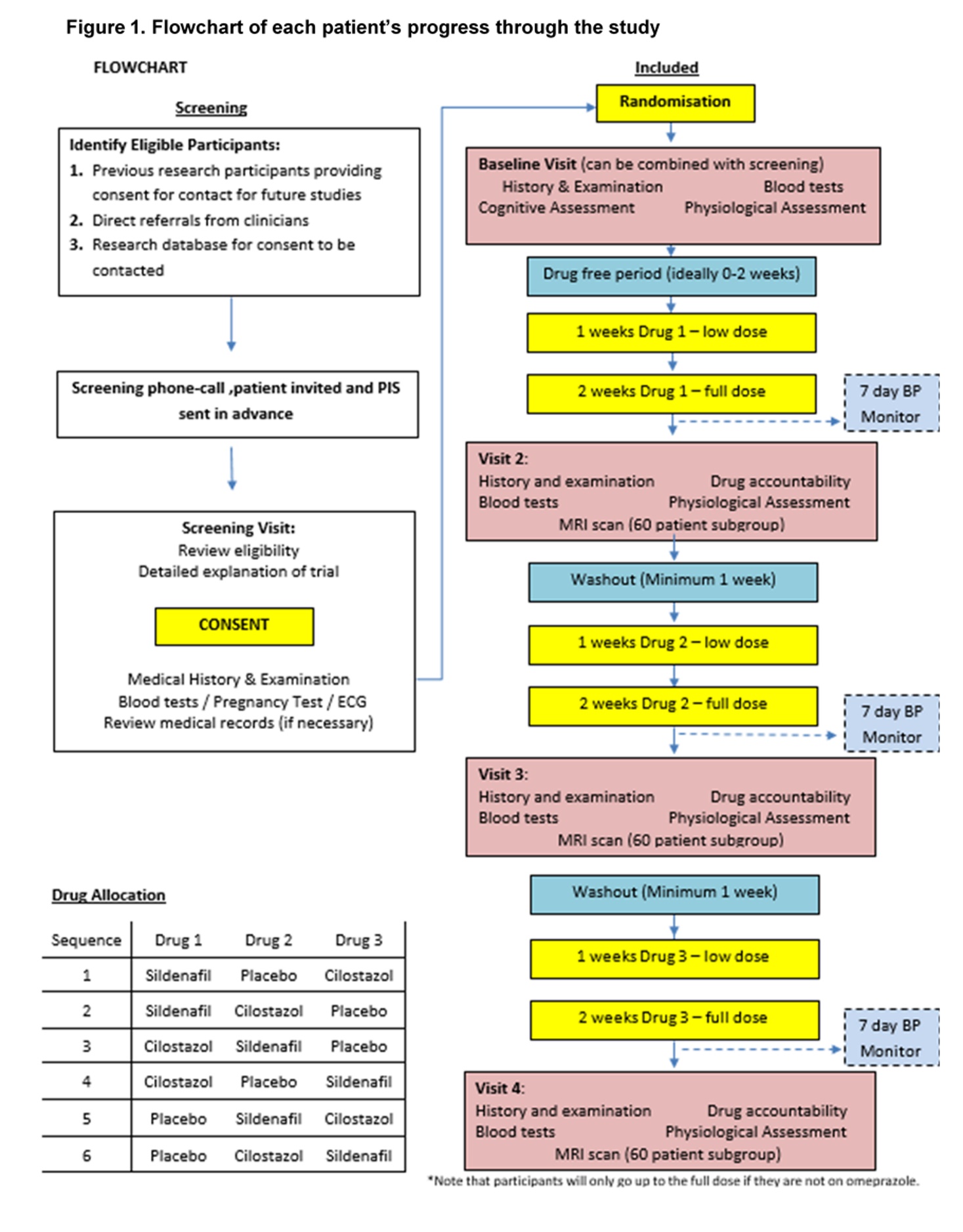


# Figure S2. Bivariate correlations and distributions of key indices.

# Scatter plots in the bottom left show the linear correlation between the index on each row versus each column, with the corresponding r value in the top right. The histogram for the distribution of each variable is shown in the diagonal. MFV=mean flow velocity; PI=pulsatility index; CVCi=cerebrovascular conductance index; RAP=resistance area product;CVR=cerebrovascular reactivity;WMH=white matter hyperintensities; NAWM= normal appearing white matter; GM=grey matter;perf=perfusion.


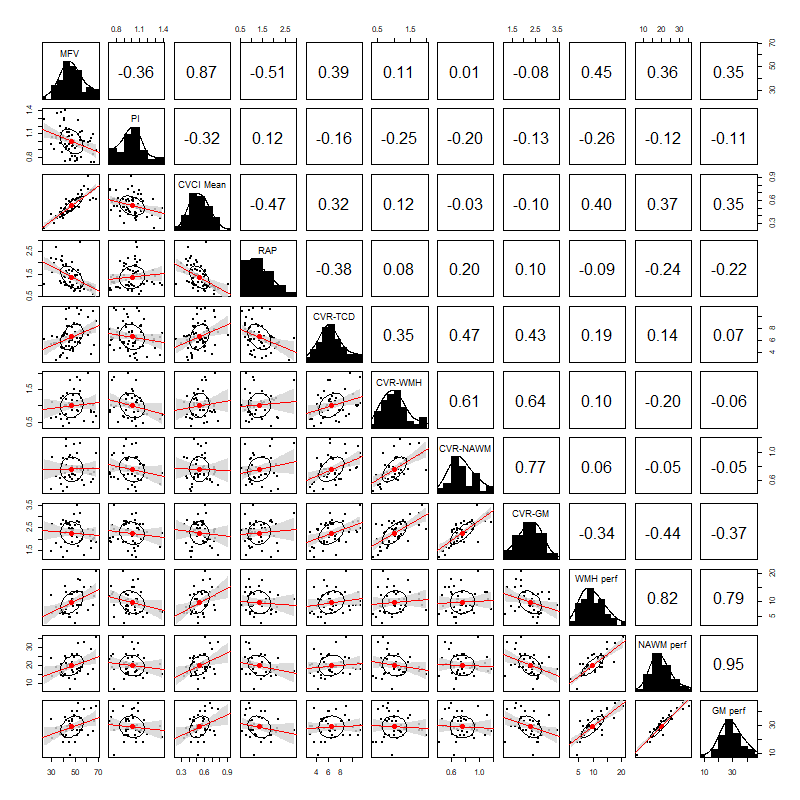


# Table S1. Derivation of key variables

| Variable | Technique | Measurement | Calculation |
| --- | --- | --- | --- |
| Aortic SBP | Applanation tonometry | Series of radial artery pulse waveforms | Standardised transfer function |
| Aortic DBP | Applanation tonometry | Series of radial artery pulse waveforms | Standardised transfer function |
| Peak systolic velocity | TCD | Manual measurement from 3 TCD waveforms | Mean of 3 measures |
| End diastolic velocity | TCD | Manual measurement from 3 TCD waveforms | Mean of 3 measures |
| Mean velocity | TCD | Derived from 3 TCD waveforms | EDV + (PSV – EDV)/3 |
| Pulsatility index | TCD | Derived from 3 TCD waveforms | (PSV – EDV) / MFV |
| CVR-TCD PSV | TCD / BP | Derived from peaks of TCD waveforms vs end-tidal CO2 (concurrent) | Unstandardised beta from GLM between PSV and etCO2 |
| CVR-TCD EDV | TCD / BP | Derived from troughs of TCD waveforms vs end-tidal CO2 (concurrent) | Unstandardised beta from GLM between EDV and etCO2 |
| CVR-TCD MFV | TCD / BP | Derived from mean of TCD waveforms vs end-tidal CO2 (concurrent) | Unstandardised beta from GLM between MFV and etCO2 |
| CVCi | TCD / BP | Derived from manual TCD and aortic BPs | MFV / Aortic MBP |
| RAP | TCD / BP | Derived from continuous TCD envelope and continuous BP | Inverse of beta-coefficient (linear regression) for BP vs TCD in diastolic phase of cardiac cycle |
| CVR WMH | MRI | Derived from % BOLD signal versus first 90s, averaged over WMH voxels, and etCO2 | Beta-coefficient for linear regression between %BOLD and etCO2 in FEAT-FSL |
| CVR NAWM | MRI | As for CVR-WMH but within NAWM | As for CVR-WMH |
| CVR GM | MRI | As for CVR-WMH but within grey matter | As for CVR-WMH |
| MRI perf WMH | MRI | pcASL derived perfusion | Averaged across WMH |
| MRI perf NAWM | MRI | pcASL derived perfusion | Averaged across NAWM |
| MRI perf GM | MRI | pcASL derived perfusion | Averaged across GM |

# Table S2. Associations between alternative TCD markers with demographic risk factors

| Variables | PSV  r2 | PSV  p-val | EDV  r2 | EDV  p-val | PI  r2 | PI  p-val | MCA CVR PSV r2 | MCA CVR PSV p | MCA CVR EDV r2 | MCA CVR EDV p | RAP  r2 | RAP  p-val |
| --- | --- | --- | --- | --- | --- | --- | --- | --- | --- | --- | --- | --- |
| Age | 0.012 | 0.351 | 0.193 | <0.001 | 0.403 | <0.001 | 0.009 | 0.428 | 0.051 | 0.06 | 0.083 | 0.022 |
| Male | 0.061 | 0.035 | 0.026 | 0.172 | 0.002 | 0.735 | 0.096 | 0.009 | 0.089 | 0.012 | 0.005 | 0.601 |
| Diabetes | 0.021 | 0.219 | 0.036 | 0.108 | 0.018 | 0.259 | 0.018 | 0.271 | 0.016 | 0.302 | 0.05 | 0.077 |
| Hypertension | 0.005 | 0.534 | 0 | 0.962 | 0.015 | 0.307 | 0.004 | 0.621 | 0.006 | 0.515 | 0.002 | 0.726 |
| Smoker | 0.408 | 0.176 | 0.528 | 0.24 | 0.521 | 0.907 | 0.587 | 0.124 | 0.647 | 0.009 | 0.482 | 0.053 |
| Alcohol | 0.32 | 0.988 | 0.313 | 0.444 | 0.334 | 0.193 | 0.233 | 0.599 | 0.212 | 0.964 | 0.246 | 0.846 |
| Systolic BP | 0.001 | 0.792 | 0.013 | 0.336 | 0.085 | 0.012 | 0.003 | 0.647 | 0.004 | 0.6 | 0.048 | 0.085 |
| Diastolic BP | 0.002 | 0.701 | 0.005 | 0.55 | 0.021 | 0.224 | 0.007 | 0.478 | 0.007 | 0.504 | 0.001 | 0.845 |
| Stroke | 0.053 | 0.049 | 0.01 | 0.406 | 0.026 | 0.175 | 0.041 | 0.093 | 0.063 | 0.036 | 0.069 | 0.038 |
| MoCA | 0.047 | 0.065 | 0.075 | 0.019 | 0.038 | 0.099 | 0.029 | 0.158 | 0.037 | 0.113 | 0.087 | 0.019 |
| WMH volume | 0.102 | 0.017 | 0.192 | 0.001 | 0.141 | 0.004 | 0.089 | 0.032 | 0.112 | 0.015 | 0.113 | 0.016 |

#

# Table S3. Associations between alternative MRI markers with demographic risk factors

| Variables | GM CVR r2 | GM CVR p | Brainstem CVR r2 | Brainstem CVR p | NAWM perfusion r2 | NAWM perfusion p | WMH perfusion r2 | WMH perfusion p | Brainstem perfusion r2 | Brainstem perfusion p |
| --- | --- | --- | --- | --- | --- | --- | --- | --- | --- | --- |
| Age | 0.017 | 0.386 | 0.101 | 0.03 | 0 | 0.963 | 0.073 | 0.079 | 0 | 0.938 |
| Male | 0.023 | 0.314 | 0.004 | 0.664 | 0.14 | 0.013 | 0.038 | 0.212 | 0.203 | 0.002 |
| Diabetes | 0 | 0.947 | 0.016 | 0.392 | 0.039 | 0.206 | 0.051 | 0.144 | 0.03 | 0.27 |
| Hypertension | 0.018 | 0.366 | 0.03 | 0.241 | 0.076 | 0.073 | 0.072 | 0.081 | 0.066 | 0.096 |
| Smoker | 0.527 | 0.832 | 0.497 | 0.723 | 0.536 | 0.297 | 0.486 | 0.602 | 0.5 | 0.5 |
| Alcohol | 0.411 | 0.125 | 0.419 | 0.153 | 0.547 | 0.694 | 0.563 | 0.345 | 0.545 | 0.887 |
| Systolic BP | 0.012 | 0.457 | 0.041 | 0.174 | 0 | 0.984 | 0.001 | 0.866 | 0 | 0.955 |
| Diastolic BP | 0.03 | 0.244 | 0.015 | 0.42 | 0.012 | 0.488 | 0.001 | 0.859 | 0.03 | 0.268 |
| Stroke | 0.011 | 0.482 | 0.014 | 0.43 | 0.017 | 0.402 | 0 | 0.97 | 0.019 | 0.379 |
| MoCA | 0.041 | 0.172 | 0.003 | 0.709 | 0.048 | 0.159 | 0.047 | 0.161 | 0.068 | 0.091 |
| WMH volume | 0.025 | 0.292 | 0 | 0.945 | 0.064 | 0.106 | 0.169 | 0.007 | 0.079 | 0.072 |

#

# Table S4. Standardised associations between key markers and demographic risk factors

| Variables | MFV r2 | MFV p | CVCi r2 | CVCi p | MCA CVR r2 | MCA CVR p | WMH CVR r2 | WMH CVR p | NAWM CVR r2 | NAWM CVR p | GM perfusion r2 | GM perfusion p-val |
| --- | --- | --- | --- | --- | --- | --- | --- | --- | --- | --- | --- | --- |
| Age | 0.082 | 0.014 | 0.038 | 0.095 | 0.016 | 0.292 | 0.18 | 0.003 | 0.035 | 0.205 | 0.002 | 0.758 |
| Male | 0.046 | 0.069 | 0.119 | 0.002 | 0.128 | 0.002 | 0.018 | 0.371 | 0.01 | 0.494 | 0.11 | 0.03 |
| Diabetes | 0.031 | 0.139 | 0.033 | 0.12 | 0.015 | 0.312 | 0.002 | 0.78 | 0.016 | 0.404 | 0.06 | 0.112 |
| Hypertension | 0.001 | 0.764 | 0 | 0.958 | 0.013 | 0.341 | 0.001 | 0.812 | 0.008 | 0.559 | 0.052 | 0.142 |
| Smoker | 0.474 | 0.183 | 0.452 | 0.309 | 0.613 | 0.057 | 0.445 | 0.872 | 0.582 | 0.834 | 0.526 | 0.523 |
| Alcohol | 0.313 | 0.683 | 0.306 | 0.221 | 0.273 | 0.984 | 0.528 | 0.377 | 0.358 | 0.115 | 0.566 | 0.603 |
| Systolic BP | 0.002 | 0.717 | 0.145 | 0.001 | 0.006 | 0.538 | 0.036 | 0.2 | 0.003 | 0.696 | 0 | 0.996 |
| Diastolic BP | 0 | 0.912 | 0.117 | 0.003 | 0 | 0.955 | 0.009 | 0.518 | 0.037 | 0.196 | 0.008 | 0.557 |
| Stroke | 0.03 | 0.145 | 0.017 | 0.259 | 0.081 | 0.017 | 0.071 | 0.071 | 0.009 | 0.532 | 0.006 | 0.635 |
| MoCA | 0.066 | 0.029 | 0.052 | 0.049 | 0.039 | 0.1 | 0.011 | 0.479 | 0.033 | 0.224 | 0.073 | 0.08 |
| WMH volume | 0.155 | 0.003 | 0.106 | 0.013 | 0.093 | 0.028 | 0.146 | 0.009 | 0 | 0.885 | 0.082 | 0.065 |

#

# Table S5. Adjusted associations between key markers and demographic risk factors. Beta-coefficients are standardised. Adjusted for age, sex and cardiovascular risk factors.

| Variables | MFV r2 | MFV p | CVCi r2 | CVCi p | MCA CVR r2 | MCA CVR p | WMH CVR r2 | WMH CVR p | NAWM CVR r2 | NAWM CVR p | GM perfusion r2 | GM perfusion p-val |
| --- | --- | --- | --- | --- | --- | --- | --- | --- | --- | --- | --- | --- |
| Age | 0.148 | 0.005 | 0.177 | 0.026 | 0.153 | 0.16 | 0.19 | 0.004 | 0.043 | 0.227 | 0.114 | 0.647 |
| Male | 0.148 | 0.024 | 0.177 | 0.001 | 0.153 | 0.002 | 0.19 | 0.467 | 0.043 | 0.556 | 0.114 | 0.03 |
| Diabetes | 0.159 | 0.339 | 0.188 | 0.332 | 0.157 | 0.601 | 0.191 | 0.914 | 0.061 | 0.376 | 0.151 | 0.2 |
| Hypertension | 0.164 | 0.249 | 0.185 | 0.406 | 0.181 | 0.138 | 0.229 | 0.147 | 0.068 | 0.289 | 0.142 | 0.266 |
| Smoker | 0.528 | 0.38 | 0.564 | 0.376 | 0.65 | 0.13 | 0.616 | 0.172 | 0.603 | 0.861 | 0.573 | 0.413 |
| Alcohol | 0.376 | 0.812 | 0.396 | 0.292 | 0.484 | 0.644 | 0.6 | 0.885 | 0.406 | 0.3 | 0.615 | 0.839 |
| Systolic BP | 0.148 | 0.949 | 0.295 | 0.001 | 0.165 | 0.335 | 0.206 | 0.361 | 0.044 | 0.809 | 0.119 | 0.661 |
| Diastolic BP | 0.148 | 0.958 | 0.276 | 0.003 | 0.166 | 0.321 | 0.191 | 0.9 | 0.062 | 0.355 | 0.115 | 0.92 |
| Stroke | 0.191 | 0.059 | 0.191 | 0.284 | 0.221 | 0.019 | 0.217 | 0.234 | 0.044 | 0.805 | 0.114 | 0.952 |
| MoCA | 0.165 | 0.228 | 0.186 | 0.372 | 0.162 | 0.407 | 0.191 | 0.853 | 0.092 | 0.136 | 0.144 | 0.251 |
| WMH volume | 0.232 | 0.104 | 0.291 | 0.382 | 0.267 | 0.144 | 0.272 | 0.027 | 0.066 | 0.589 | 0.147 | 0.234 |

#

# Table S6. Correlations between markers of cerebral perfusion with alternative physiological indices. Beta-coefficients are standardised

| Variables | MFV beta | MFV r2 | MFV p | GM perf beta | GM perf r2 | GM perf p | NAWM perf beta | NAWM perf r2 | NAWM perf p |
| --- | --- | --- | --- | --- | --- | --- | --- | --- | --- |
| Aortic SBP | -0.043 | 0.002 | 0.717 | 0.001 | 0.000 | 0.996 | 0.003 | 0.000 | 0.984 |
| Aortic DBP | 0.013 | 0.000 | 0.912 | -0.103 | 0.008 | 0.557 | -0.122 | 0.012 | 0.488 |
| Aortic MBP | -0.016 | 0.000 | 0.894 | -0.058 | 0.003 | 0.735 | -0.067 | 0.004 | 0.695 |
| PSV | 0.958 | 0.918 | 0.000 | 0.345 | 0.110 | 0.030 | 0.355 | 0.116 | 0.025 |
| EDV | 0.957 | 0.917 | 0.000 | 0.367 | 0.112 | 0.028 | 0.385 | 0.124 | 0.021 |
| MFV | 1.000 | 1.000 | 0.000 | 0.374 | 0.123 | 0.021 | 0.389 | 0.133 | 0.016 |
| PI | -0.345 | 0.119 | 0.003 | -0.119 | 0.012 | 0.489 | -0.128 | 0.014 | 0.455 |
| CVR MCA PSV | 0.589 | 0.343 | 0.000 | 0.095 | 0.011 | 0.507 | 0.162 | 0.032 | 0.256 |
| CVR MCA EDV | 0.596 | 0.355 | 0.000 | 0.086 | 0.010 | 0.538 | 0.125 | 0.020 | 0.370 |
| CVR MCA MFV | 0.593 | 0.342 | 0.000 | 0.064 | 0.006 | 0.637 | 0.117 | 0.018 | 0.394 |
| CVCi MFV | 0.929 | 0.812 | 0.000 | 0.396 | 0.120 | 0.023 | 0.419 | 0.135 | 0.015 |
| CVCi PSV | 0.868 | 0.727 | 0.000 | 0.305 | 0.077 | 0.071 | 0.313 | 0.081 | 0.065 |
| CVCi EDV | 0.932 | 0.798 | 0.000 | 0.444 | 0.148 | 0.011 | 0.477 | 0.170 | 0.006 |
| RAP DBP | -0.514 | 0.225 | <0.001 | -0.220 | 0.049 | 0.177 | -0.226 | 0.058 | 0.138 |
| MRI CVR WMH | 0.114 | 0.013 | 0.444 | -0.056 | 0.003 | 0.716 | -0.193 | 0.039 | 0.205 |
| MRI CVR NAWM | 0.012 | 0.000 | 0.937 | -0.046 | 0.002 | 0.761 | -0.046 | 0.002 | 0.763 |
| MRI CVR GM | -0.090 | 0.008 | 0.545 | -0.340 | 0.123 | 0.021 | -0.415 | 0.184 | 0.004 |
| MRI CVR Brainstem | 0.037 | 0.001 | 0.803 | -0.159 | 0.026 | 0.298 | -0.203 | 0.043 | 0.182 |
| MRI perf WMH | 0.424 | 0.205 | 0.002 | 0.794 | 0.631 | 0.000 | 0.820 | 0.673 | 0.000 |
| MRI perf NAWM | 0.341 | 0.133 | 0.016 | 0.950 | 0.902 | 0.000 | 1.000 | 1.000 | 0.000 |
| MRI perf GM | 0.329 | 0.123 | 0.021 | 1.000 | 1.000 | 0.000 | 0.950 | 0.902 | 0.000 |
| MRI perf Brainstem | 0.292 | 0.097 | 0.042 | 0.897 | 0.805 | 0.000 | 0.920 | 0.846 | 0.000 |

# Table S7. Correlation between drug-effects (sildenafil vs placebo) on cerebrovascular endothelial dysfunction with correlated markers

| Variables | MFV CVR beta | MFV CVR r2 | MFV CVR p | WMH CVR beta | WMH CVR r2 | WMH CVR p | NAWM CVR beta | NAWM CVR r2 | NAWM CVR p |
| --- | --- | --- | --- | --- | --- | --- | --- | --- | --- |
| Aortic SBP | 0.018 | 0.000 | 0.887 | -0.183 | 0.038 | 0.210 | 0.102 | 0.012 | 0.486 |
| Aortic DBP | 0.100 | 0.010 | 0.442 | 0.051 | 0.003 | 0.735 | 0.183 | 0.037 | 0.218 |
| Aortic MBP | 0.069 | 0.005 | 0.596 | -0.063 | 0.005 | 0.664 | 0.154 | 0.027 | 0.289 |
| PSV | 0.256 | 0.066 | 0.045 | 0.249 | 0.059 | 0.125 | 0.100 | 0.010 | 0.542 |
| EDV | 0.302 | 0.092 | 0.018 | 0.234 | 0.061 | 0.119 | 0.073 | 0.006 | 0.633 |
| MFV | 0.293 | 0.087 | 0.021 | 0.249 | 0.065 | 0.107 | 0.088 | 0.008 | 0.574 |
| PI | -0.176 | 0.031 | 0.174 | -0.214 | 0.036 | 0.238 | -0.103 | 0.008 | 0.573 |
| CVR MCA PSV | 0.936 | 0.876 | 0.000 | 0.216 | 0.048 | 0.163 | 0.117 | 0.014 | 0.455 |
| CVR MCA EDV | 0.970 | 0.940 | 0.000 | 0.242 | 0.058 | 0.124 | 0.040 | 0.002 | 0.802 |
| CVR MCA MFV | 1.000 | 1.000 | 0.000 | 0.242 | 0.059 | 0.121 | 0.058 | 0.003 | 0.716 |
| CVCi MFV | 0.222 | 0.050 | 0.079 | 0.190 | 0.046 | 0.169 | -0.037 | 0.002 | 0.790 |
| CVCi PSV | 0.185 | 0.034 | 0.148 | 0.218 | 0.061 | 0.111 | -0.017 | 0.000 | 0.901 |
| CVCi EDV | 0.246 | 0.063 | 0.048 | 0.161 | 0.032 | 0.250 | -0.038 | 0.002 | 0.786 |
| RAP DBP | -0.209 | 0.042 | 0.125 | -0.207 | 0.042 | 0.194 | -0.114 | 0.013 | 0.477 |
| MRI CVR WMH | 0.244 | 0.059 | 0.121 | 1.000 | 1.000 | 0.000 | 0.746 | 0.556 | 0.000 |
| MRI CVR NAWM | 0.058 | 0.003 | 0.716 | 0.746 | 0.556 | 0.000 | 1.000 | 1.000 | 0.000 |
| MRI CVR GM | 0.082 | 0.007 | 0.608 | 0.688 | 0.473 | 0.000 | 0.776 | 0.603 | 0.000 |
| MRI CVR Brainstem | 0.017 | 0.000 | 0.919 | 0.564 | 0.318 | 0.000 | 0.787 | 0.619 | 0.000 |
| MRI perf WMH | 0.352 | 0.155 | 0.023 | 0.192 | 0.036 | 0.285 | 0.148 | 0.020 | 0.426 |
| MRI perf NAWM | 0.243 | 0.073 | 0.128 | -0.041 | 0.002 | 0.823 | 0.011 | 0.000 | 0.953 |
| MRI perf GM | 0.276 | 0.094 | 0.083 | 0.068 | 0.004 | 0.709 | 0.070 | 0.004 | 0.707 |
| MRI perf Brainstem | 0.271 | 0.088 | 0.094 | 0.081 | 0.006 | 0.656 | 0.141 | 0.018 | 0.449 |

# Table S8. Correlations between drug-effects (sildenafil-placebo) on cerebral perfusion with alternative physiological indices

| Variables | MFV beta | MFV r2 | MFV p | GM perf beta | GM perf r2 | GM perf p | NAWM perf beta | NAWM perf r2 | NAWM perf p |
| --- | --- | --- | --- | --- | --- | --- | --- | --- | --- |
| Aortic SBP | 0.002 | 0.012 | 0.486 | 0.017 | 0.002 | 0.810 | 0.012 | 0.002 | 0.824 |
| Aortic DBP | 0.005 | 0.037 | 0.218 | -0.101 | 0.020 | 0.424 | -0.088 | 0.026 | 0.364 |
| Aortic MBP | 0.004 | 0.027 | 0.289 | -0.037 | 0.004 | 0.737 | -0.034 | 0.005 | 0.684 |
| PSV | 0.002 | 0.010 | 0.542 | 0.093 | 0.023 | 0.408 | 0.038 | 0.006 | 0.665 |
| EDV | 0.003 | 0.006 | 0.633 | -0.005 | 0.000 | 0.982 | -0.043 | 0.003 | 0.776 |
| MFV |  |  |  | 0.065 | 0.005 | 0.697 | 0.007 | 0.000 | 0.958 |
| PI | -0.167 | 0.008 | 0.573 | 9.675 | 0.027 | 0.366 | 6.106 | 0.019 | 0.457 |
| CVR MCA PSV | 0.006 | 0.014 | 0.455 | 0.445 | 0.069 | 0.140 | 0.278 | 0.046 | 0.231 |
| CVR MCA EDV | 0.004 | 0.002 | 0.802 | 0.959 | 0.079 | 0.114 | 0.677 | 0.067 | 0.147 |
| CVR MCA MFV | 0.005 | 0.003 | 0.716 | 0.826 | 0.094 | 0.083 | 0.558 | 0.073 | 0.128 |
| CVCi MFV | -0.068 | 0.002 | 0.790 | 5.807 | 0.015 | 0.493 | 2.525 | 0.005 | 0.698 |
| CVCi PSV | -0.024 | 0.000 | 0.901 | 3.165 | 0.008 | 0.614 | 0.823 | 0.001 | 0.864 |
| CVCi EDV | -0.081 | 0.002 | 0.786 | 7.026 | 0.016 | 0.483 | 3.720 | 0.007 | 0.628 |
| RAP DBP | 0.004 | 0 | 0.978 | -0.173 | 0.027 | 0.357 | -0.051 | 0.002 | 0.787 |
| MRI CVR WMH | 0.531 | 0.556 | 0.000 | 1.386 | 0.004 | 0.709 | -0.636 | 0.002 | 0.823 |
| MRI CVR NAWM | 1.000 | 1.000 | 0.000 | 1.902 | 0.004 | 0.707 | 0.230 | 0.000 | 0.953 |
| MRI CVR GM | 0.302 | 0.603 | 0.000 | -0.803 | 0.004 | 0.723 | -1.498 | 0.024 | 0.385 |
| MRI CVR Brainstem | 0.392 | 0.619 | 0.000 | -0.776 | 0.002 | 0.800 | -0.908 | 0.005 | 0.698 |
| MRI perf WMH | 0.008 | 0.020 | 0.426 | 1.280 | 0.650 | 0.000 | 0.905 | 0.554 | 0.000 |
| MRI perf NAWM | 0.000 | 0.000 | 0.953 | 1.242 | 0.904 | 0.000 | 1.000 | 1.000 | 0.000 |
| MRI perf GM | 0.002 | 0.004 | 0.707 | 1.000 | 1.000 | 0.000 | 0.728 | 0.904 | 0.000 |
| MRI perf Brainstem | 0.004 | 0.018 | 0.449 | 0.454 | 0.273 | 0.002 | 0.403 | 0.368 | 0.000 |

# Table S9. Correlation between drug-effects (cilostazol vs placebo) on cerebrovascular endothelial dysfunction with correlated markers

| Variables | MFV CVR beta | MFV CVR r2 | MFV CVR p | WMH CVR beta | WMH CVR r2 | WMH CVR p | NAWM CVR beta | NAWM CVR r2 | NAWM CVR p |
| --- | --- | --- | --- | --- | --- | --- | --- | --- | --- |
| Aortic SBP | -0.001 | -0.018 | 0.975 | 0.000 | -0.071 | 0.978 | 0.004 | 0.051 | 0.201 |
| Aortic DBP | -0.038 | 0.001 | 0.302 | -0.006 | -0.035 | 0.494 | 0.003 | -0.060 | 0.701 |
| Aortic MBP | -0.021 | -0.010 | 0.527 | -0.002 | -0.062 | 0.731 | 0.004 | -0.013 | 0.382 |
| PSV | 0.032 | 0.019 | 0.150 | 0.001 | -0.070 | 0.911 | -0.009 | 0.097 | 0.128 |
| EDV | 0.111 | 0.073 | 0.022 | 0.015 | 0.036 | 0.232 | 0.003 | -0.062 | 0.736 |
| MFV | 0.072 | 0.049 | 0.051 | 0.011 | -0.019 | 0.408 | -0.005 | -0.053 | 0.624 |
| PI | -4.928 | 0.052 | 0.046 | -0.748 | 0.098 | 0.127 | -0.574 | 0.124 | 0.099 |
| CVR MCA PSV | 0.669 | 0.875 | 0.000 | 0.032 | 0.068 | 0.170 | 0.021 | 0.054 | 0.195 |
| CVR MCA EDV | 1.248 | 0.932 | 0.000 | 0.034 | -0.022 | 0.422 | 0.010 | -0.062 | 0.732 |
| CVR MCA MFV | 1.000 | 1.000 | 0.000 | 0.018 | -0.044 | 0.554 | 0.011 | -0.051 | 0.611 |
| CVCi MFV | 5.391 | 0.047 | 0.054 | 0.407 | -0.045 | 0.562 | -0.492 | 0.004 | 0.322 |
| CVCi PSV | 1.854 | -0.004 | 0.381 | -0.039 | -0.071 | 0.926 | -0.433 | 0.092 | 0.135 |
| CVCi EDV | 8.699 | 0.108 | 0.006 | 1.325 | 0.073 | 0.161 | -0.066 | -0.071 | 0.925 |
| RAP DBP | 0.018 | -0.019 | 0.896 | -0.086 | -0.08 | 0.851 | -0.464 | -0.003 | 0.347 |
| MRI CVR WMH | 1.453 | -0.044 | 0.554 | 1.000 | 1.000 | 0.000 | 0.564 | 0.596 | 0.000 |
| MRI CVR NAWM | 1.751 | -0.051 | 0.611 | 1.104 | 0.596 | 0.000 | 1.000 | 1.000 | 0.000 |
| MRI CVR GM | 0.221 | -0.069 | 0.860 | 0.328 | 0.377 | 0.007 | 0.163 | 0.146 | 0.080 |
| MRI CVR Brainstem | 1.897 | -0.021 | 0.421 | 0.413 | 0.125 | 0.098 | 0.454 | 0.392 | 0.006 |
| MRI perf WMH | 0.057 | -0.058 | 0.733 | -0.018 | 0.034 | 0.244 | -0.005 | -0.060 | 0.653 |
| MRI perf NAWM | -0.002 | -0.067 | 0.989 | -0.014 | 0.008 | 0.310 | -0.005 | -0.058 | 0.639 |
| MRI perf GM | 0.008 | -0.066 | 0.939 | -0.011 | 0.046 | 0.217 | -0.002 | -0.067 | 0.728 |
| MRI perf Brainstem | -0.026 | -0.062 | 0.799 | 0.003 | -0.070 | 0.784 | 0.004 | -0.052 | 0.587 |

# Table S10. Correlations between drug-effects (cilostazol-placebo) on cerebral perfusion with alternative physiological indices

| Variables | MFV beta | MFV r2 | MFV p | GM perf beta | GM perf r2 | GM perf p | NAWM perf beta | NAWM perf r2 | NAWM perf p |
| --- | --- | --- | --- | --- | --- | --- | --- | --- | --- |
| Aortic SBP | 0.004 | 0.051 | 0.201 | -0.042 | -0.052 | 0.692 | -0.034 | -0.048 | 0.644 |
| Aortic DBP | 0.003 | -0.060 | 0.701 | -0.184 | -0.020 | 0.425 | -0.128 | -0.020 | 0.429 |
| Aortic MBP | 0.004 | -0.013 | 0.382 | -0.104 | -0.038 | 0.545 | -0.077 | -0.035 | 0.523 |
| PSV | -0.009 | 0.097 | 0.128 | -0.068 | -0.051 | 0.685 | -0.036 | -0.056 | 0.759 |
| EDV | 0.003 | -0.062 | 0.736 | -0.201 | -0.016 | 0.404 | -0.085 | -0.046 | 0.620 |
| MFV | -0.005 | -0.053 | 0.624 | -0.154 | -0.030 | 0.489 | -0.069 | -0.049 | 0.659 |
| PI | -0.574 | 0.124 | 0.099 | 10.668 | -0.009 | 0.371 | 3.885 | -0.048 | 0.646 |
| CVR MCA PSV | 0.021 | 0.054 | 0.195 | -0.006 | -0.067 | 0.992 | 0.009 | -0.067 | 0.981 |
| CVR MCA EDV | 0.010 | -0.062 | 0.732 | 0.048 | -0.066 | 0.960 | 0.099 | -0.065 | 0.884 |
| CVR MCA MFV | 0.011 | -0.051 | 0.611 | 0.052 | -0.066 | 0.939 | -0.007 | -0.067 | 0.989 |
| CVCi MFV | -0.492 | 0.004 | 0.322 | 3.057 | -0.060 | 0.840 | 4.470 | -0.050 | 0.674 |
| CVCi PSV | -0.433 | 0.092 | 0.135 | 4.745 | -0.048 | 0.643 | 4.080 | -0.041 | 0.570 |
| CVCi EDV | -0.066 | -0.071 | 0.925 | -3.461 | -0.060 | 0.851 | 2.090 | -0.061 | 0.872 |
| RAP DBP | -0.075 | -0.013 | 0.589 | -0.143 | -0.066 | 0.72 | 0.004 | -0.077 | 0.992 |
| MRI CVR WMH | 0.564 | 0.596 | 0.000 | -9.993 | 0.046 | 0.217 | -5.747 | 0.008 | 0.310 |
| MRI CVR NAWM | 1.000 | 1.000 | 0.000 | -4.125 | -0.067 | 0.728 | -3.846 | -0.058 | 0.639 |
| MRI CVR GM | 0.163 | 0.146 | 0.080 | -7.727 | 0.247 | 0.034 | -4.892 | 0.194 | 0.057 |
| MRI CVR Brainstem | 0.454 | 0.392 | 0.006 | -2.636 | -0.067 | 0.731 | -1.405 | -0.071 | 0.791 |
| MRI perf WMH | -0.005 | -0.060 | 0.653 | 1.465 | 0.783 | 0.000 | 1.032 | 0.787 | 0.000 |
| MRI perf NAWM | -0.005 | -0.058 | 0.639 | 1.367 | 0.916 | 0.000 | 1.000 | 1.000 | 0.000 |
| MRI perf GM | -0.002 | -0.067 | 0.728 | 1.000 | 1.000 | 0.000 | 0.674 | 0.916 | 0.000 |
| MRI perf Brainstem | 0.004 | -0.052 | 0.587 | 0.698 | 0.439 | 0.002 | 0.501 | 0.461 | 0.001 |

# Table S11. Sample sizes at different effect sizes and power

| Effect Size (%) | 5 | 5 | 5 | 10 | 10 | 10 | 10 | 10 | 10 | SD | SD | SD |
| --- | --- | --- | --- | --- | --- | --- | --- | --- | --- | --- | --- | --- |
| Design | Parallel | Parallel | Parallel | Parallel | Parallel | Parallel | Paired | Paired | Paired | Paired | Paired | Paired |
| Power | 80 | 90 | 95 | 80 | 90 | 95 | 80 | 90 | 95 | 80 | 90 | 95 |
| Aortic SBP | 100 | 132 | 163 | 26 | 34 | 42 | 52 | 68 | 84 | 15 | 19 | 23 |
| Aortic DBP | 86 | 114 | 141 | 23 | 30 | 37 | 45 | 59 | 73 | 13 | 17 | 20 |
| PSV | 234 | 311 | 385 | 60 | 79 | 98 | 119 | 158 | 195 | 32 | 41 | 51 |
| EDV | 356 | 475 | 587 | 90 | 120 | 148 | 180 | 240 | 296 | 47 | 62 | 76 |
| MFV | 258 | 344 | 425 | 66 | 87 | 108 | 131 | 174 | 215 | 35 | 45 | 56 |
| PI | 162 | 216 | 267 | 42 | 55 | 68 | 83 | 110 | 136 | 23 | 29 | 36 |
| CVR MCA PSV | 732 | 979 | 1,211 | 184 | 246 | 304 | 368 | 492 | 607 | 94 | 125 | 154 |
| CVR MCA EDV | 619 | 828 | 1,024 | 156 | 208 | 257 | 311 | 416 | 514 | 80 | 106 | 130 |
| CVR MCA MFV | 637 | 851 | 1,053 | 160 | 214 | 265 | 320 | 428 | 529 | 82 | 109 | 134 |
| CVCi MFV | 379 | 506 | 626 | 96 | 128 | 158 | 191 | 255 | 315 | 50 | 66 | 81 |
| CVCi PSV | 326 | 435 | 537 | 83 | 110 | 136 | 165 | 220 | 271 | 43 | 57 | 70 |
| CVCi EDV | 412 | 551 | 681 | 104 | 139 | 172 | 208 | 277 | 343 | 54 | 71 | 87 |
| RAP | 784 | 1,048 | 1,295 | 197 | 263 | 325 | 394 | 526 | 650 | 100 | 133 | 164 |
| MRI CVR WMH | 908 | 1,214 | 1,502 | 228 | 305 | 377 | 456 | 609 | 753 | 116 | 154 | 190 |
| MRI CVR NAWM | 316 | 421 | 521 | 80 | 107 | 132 | 160 | 213 | 263 | 42 | 55 | 67 |
| MRI CVR GM | 352 | 470 | 581 | 89 | 119 | 147 | 178 | 237 | 293 | 46 | 61 | 75 |
| MRI perf WMH | 1,027 | 1,373 | 1,698 | 258 | 345 | 426 | 515 | 689 | 851 | 131 | 174 | 215 |
| MRI perf NAWM | 530 | 707 | 875 | 133 | 178 | 220 | 266 | 356 | 440 | 68 | 91 | 112 |
| MRI perf GM | 433 | 578 | 714 | 109 | 146 | 180 | 218 | 291 | 359 | 56 | 75 | 92 |
